# The Modus Operandi of Various Kinds of Baths, Sea-Bathing, Heat and Cold, Physiologically Explained

**Published:** 1861-07

**Authors:** 


The Modus Operandi of Various Kinds of Baths, Sea-Bathing, Heat and Cold, Physiologically Explained.—By John O'Reilly, M. D., Licentiate and Fellow of the Royal College of Surgeons in Ireland; Resident Fellow of the New York Academy of Medicine; Member of the Medico-Chirugical College of New York; late Medical Officer to the Oldcastle Workhouse and Fever Hospital, Ireland. New York: Hall, Clayton & Co., Printers, 46 Pine Street. 1861.

In the course of a dozen or so pages Dr. O'Reilly manages to discuss the *modus operandi* and good effects of the warm bath, its use in preventing fever and inflammation, and as a remedy in strangulated hernia, the bad effects of ice, remarks on hernia, fainting caused by the warm bath, mode of resuscitation, Marshall Hall's method of treating asphyxiated infants, the mode in which infants born apparently dead are restored to life, efficacy of cold douche in post-partum hæmorrhage, mode of action of ergot of rye in arresting hæmorrhage, how sprinkling an infant with cold water resuscitates it, how cold air or ice arrests hæmorrhage after an operation when oozing of blood continues, wounds dressed immediately after an operation, good effects of cold douche in fever and encephalitis, how a drink of cold water causes death, state of a wound after exposure for three hours, Mr. Liston's remarks, Mr. Macartney's theory, Sir Astley Cooper's ideas, Mr. Hunter's views, cold water dressing, the mode of treatment of wounds after operations, effects of the cold bath, the cause of spasms by cold, by tetanus, by strychnine, by lead, by Asiatic cholera, treatment of spasms produced by cold water, *modus operandi* of salt-water bathing, phosphorus, chloride of sodium, good effects of cold baths, shower baths, cause of suspended animation, *modus operandi* of sulphur baths, iodine baths, nitro-muriatic acid baths, and of iodine in Bright's disease.

He also adds five notes on as many more subjects; so that it will be seen this pamphlet of pp. 23, is an exceedingly comprehensive affair.

